# Reverse engineering and analysis of large genome-scale gene networks

**DOI:** 10.1093/nar/gks904

**Published:** 2012-10-05

**Authors:** Maneesha Aluru, Jaroslaw Zola, Dan Nettleton, Srinivas Aluru

**Affiliations:** ^1^Department of Genetics, Development, and Cell Biology, ^2^Department of Electrical and Computer Engineering, ^3^Department of Statistics, Iowa State University, Ames, IA 50011, USA and ^4^Department of Computer Science and Engineering, Indian Institute of Technology Bombay, Powai, Mumbai 400076, India

## Abstract

Reverse engineering the whole-genome networks of complex multicellular organisms continues to remain a challenge. While simpler models easily scale to large number of genes and gene expression datasets, more accurate models are compute intensive limiting their scale of applicability. To enable fast and accurate reconstruction of large networks, we developed Tool for Inferring Network of Genes (TINGe), a parallel mutual information (MI)-based program. The novel features of our approach include: (i) B-spline-based formulation for linear-time computation of MI, (ii) a novel algorithm for direct permutation testing and (iii) development of parallel algorithms to reduce run-time and facilitate construction of large networks. We assess the quality of our method by comparison with ARACNe (Algorithm for the Reconstruction of Accurate Cellular Networks) and GeneNet and demonstrate its unique capability by reverse engineering the whole-genome network of *Arabidopsis thaliana* from 3137 Affymetrix ATH1 GeneChips in just 9 min on a 1024-core cluster. We further report on the development of a new software Gene Network Analyzer (GeNA) for extracting context-specific subnetworks from a given set of seed genes. Using TINGe and GeNA, we performed analysis of 241 *Arabidopsis* AraCyc 8.0 pathways, and the results are made available through the web.

## INTRODUCTION

Genes act together in networks to execute various cellular functions in response to both endogenous (e.g. developmental) and exogenous (e.g. light) stimuli. The elucidation of these complex inter-gene interactions is fundamental to accelerating the pace of novel biological discoveries. With the wide adoption of microarray technology and more recently RNA-seq, public repositories containing large number of gene expression profiles are readily available, spurring the development of numerous computational methods for gene network inference. Techniques developed include Pearson correlation (1,2), graphical Gaussian modeling (GGM) (3–5), information theory (6,7), Bayesian networks (8,9), entropy maximization (10), singular value decomposition (11) and message passing algorithms (12), among many others. Despite this intense research, inferring genome-scale gene networks of complex organisms (e.g. plants and mammals) and analyzing such networks to extract biologically valid hypotheses remain important challenges in systems biology.

Two key problems remain with the current methods for reverse engineering gene networks. One is the quality of network inference and the ability to predict complex gene interactions, and distinguish indirect interactions from direct ones (13). In a recent comprehensive study of 29 network inference methods, Marbach *et al.* (14) concluded that many do poorly on an absolute basis and 11 do no better than random guessing. A second challenge is to scale inference methods to organisms with tens of thousands of genes and large number of experiments. The computational cost of network inference grows at least as square of the number of the genes, and at least linearly with the number of experiments analyzed. Often, sophisticated methods that model non-linear interactions such as information-theoretic and Bayesian approaches are compute-intensive, further straining the scaling limitations. In addition, statistical techniques such as permutation testing and bootstrapping add an extra layer of computational complexity. As a result, current approaches compromise on either network/data sizes or the inference method. For example, Pearson correlation is a popular method used to build large gene networks. Although it is faster to compute gene co-expressions using Pearson correlation, this approach can infer only linear relationships and is unable to distinguish indirect interactions from direct ones. When using sophisticated approaches, gene networks are often built piecemeal on many smaller subsets of genes and subsequently combined into a larger network (3,15,16), which may result in missing interactions and sampling bias.

Our goal is to remove computational feasibility driven limitations on number of genes or expression profiles, while choosing computational and statistical protocols for inference accuracy rather than computational expediency. Here, we present a novel approach to reverse engineer genome-scale gene networks from large number of expression profiles based on mutual information (MI), data processing inequality (DPI) and permutation testing to assess statistical significance of each inferred edge. While many of these concepts are known, we present algorithmic improvements and develop a parallel method for enabling inference of large networks. We demonstrate the utility of our method by inferring the whole-genome network of the model plant *Arabidopsis thaliana* from all available Affymtrix ATH1 GeneChip experiments in just 9 min on a 1024-core cluster. Thus, our method provides a valuable tool to researchers to directly construct genome-scale networks from a large number of gene expression profiles using well-regarded computational and statistical approaches, and at unprecedented speeds.

Although complex, network inference is just a preliminary step that must be followed by some type of network analysis to extract information of biological significance. Towards this end, many different techniques including basic microarray or graph clustering methods (17,18), graph theoretic approaches (19–21), integrated strategies that combine differential gene expression with network data (22–26), and literature-based inference (27), have been employed. Often, biological pathways are partially characterized based on decades of laboratory research. To gain further understanding, the known genes are taken as seeds and their graph neighborhood extracted. One class of methods select genes at a distance of one or two edges from a seed gene, or those that lie on shortest paths between pairs of seed genes (19). When applied to large genome-scale gene networks, these approaches yielded subnetworks that are too large to be pertinent. Recently, a second class of methods emerged that are motivated by the success of page ranking methods in determining relevant pages on large world wide web graphs (28). Ranking-based methods were designed in the context of interpreting microarray experiments for identifying and ranking differentially expressed genes (21), and in prioritizing disease genes using protein–protein interaction networks (29–32). In this work, we adopt a similar approach to develop a subnetwork extraction method. The method extracts subnetworks containing given seed genes by including additional genes that are ranked highly in terms of their relative importance to seed genes, taking into account the network topology and MI between pairs of genes.

In this work, we provide three resources for the scientific community. We make available two open-source programs—Tool for Inferring Network of Genes (TINGe) and Gene Network Analyzer (GeNA)—that can be used for gene network inference and subnetwork extraction. GeNA interfaces with Cytoscape, a dominantly used program for visualization and analysis of molecular networks (33). In addition, we report on the construction of the *Arabidopsis* whole-genome network using TINGe, and its analysis with GeNA using various cellular processes and metabolic pathways as guide(s). The whole-genome network and our analysis of all 241 *Arabidopsis* AraCyc 8.0 pathways each containing at least three experimentally verified genes is made available for investigation by other researchers.

## MATERIALS AND METHODS

### Datasets

We collected a total of 3546 non-redundant Affymetrix *Arabidopsis* ATH1 expression profiles from NASC, AtGenExpress, ArrayExpress and GEO public repositories (Table 1 and Supplementary Table S1). These are grouped into 197 experiments and include steady-state and time-series data generated from various tissues and organs, and under different developmental, treatment and environmental conditions.

**Table 1.** Microarray data acquisition

Database	Experiments	CEL files	QC filtered
ArrayExpress	42	816	44
AtGenExpress	44	1334	289
GEO	60	859	59
NASC	51	537	17
Total	197	3546	409

List of databases, number of experiments obtained from each database and the number of original CEL files that passed quality control procedures.

### Quality control

The data were screened for several measures typical to the Affymetrix platform (34). Using the simpleaffy package from Bioconductor, we inspected scale factors and presence of BioB spike-in transcript. Chips that fell outside of 3-fold of the mean scale factor for a given experiment, or did not call BioB present, were removed. To detect outlier chips within an experiment, we used the affyPLM Bioconductor library. This uses the RMA probe-level model that reports relative log expression and normalized unscaled standard errors. These measures should be centered around zero and one, respectively, and should have a small spread. We removed chips with interquartile range (IQR) higher than 0.75 and that were >0.075 from the required center. A total of 3137 chips survived this process.

### Normalization

The goal of this stage is to render gene expressions comparable across experiments. We converted raw Affymetrix probe intensities into expression values using the standard MAS 5.0 procedure with a scaling factor of 1000. Subsequently, expression measures were transformed to 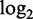 space and changed to 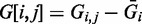, where 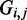 is the raw expression value of gene *i* in chip *j*, 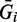 is the average expression of gene *i* across all the chips in the experiment containing chip *j* and *G*[*i, j*] is the normalized expression of gene *i* in chip *j*. Finally, quantile normalization was performed using the limma R package.

### Data filtering and annotation

To estimate MI based on expression profiles, it is important they cover wide range of expression. Based on empirical analysis, we removed probe-sets with expression profile IQR <0.65. The last stage is to create a correct mapping between probe-sets and genes. Due to evolving changes in *Arabidopsis* annotation, many probe-sets match multiple genes and vice versa. Based on annotation files available from Affymetrix and TAIR, we created an initial map that contained all 22 810 probe-sets, out of which 215 were characterized as ‘no_match’ (i.e. those with no corresponding *Arabidopsis* gene identifier (AGI)). From this map, we removed probe-sets that were mapping to more than three AGIs as we believe that such probes are not able to provide expression signal that would be useful in co-expression analysis. Next, we ran a clustering algorithm that placed two probe-sets in the same cluster if they shared one or more AGIs. For each such cluster, a probe-set that mapped to the fewest number of AGIs was selected as a representative and was preserved in the final dataset while all other probe-sets from the cluster were removed. As a result, we obtained the final expression matrix with 3137 observations and 15 596 probe-sets mapping to 15 495 genes.

### Parallel method

**Figure 1.**
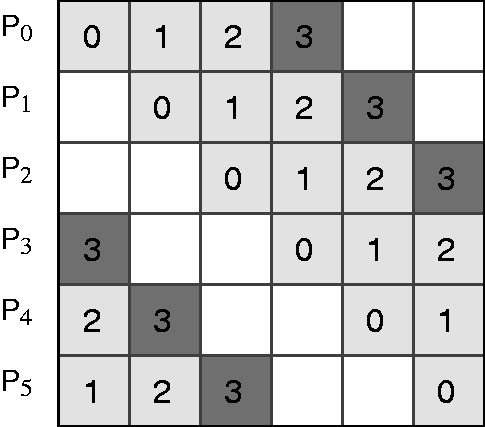
The network to be inferred is represented as an 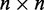 adjacency matrix *D*, where *n* is the number of genes. The matrix is partitioned into 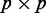 blocks of submatrices as shown. Each processor is assigned a row of submatrices. The number inside a submatrix indicates the stage at which the submatrix is computed. Only half the matrix is computed as it is symmetric.

Let *G* denote the 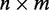 gene expression matrix, where *G*[*i, j*] contains normalized expression value of gene *i* in chip *j*. We compute an 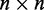 adjacency matrix *D* corresponding to inferred network *N* such that for each edge (*i, j*) in *N*, *D*[*i, j*] contains the MI-value between gene expression profiles *G*[*i*, 1 … *m*] and *G*[*j*, 1 … *m*]. Initially, we compute MI for all pairs and record in *D*. In our case, *G* has over 45 million expression values and *D* has over 240 million MI-values. Apart from parallelizing computations, it is important to distribute the matrices and not replicate them on each processor. Let *P*-value denote the number of processors each with a unique identifying rank in the range 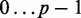. For simplicity, assume *P*-value divides *n*. *G* is partitioned so that processor *i* has rows 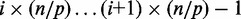 in its memory. *D* is partitioned similarly for storage purposes, but is partitioned into 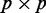 blocks of submatrices 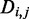 (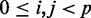) of size 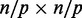 for computation purposes (Figure 1). Note that *D* is a symmetric matrix, requiring computation of only half the entries. The parallel algorithm proceeds in 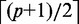 stages. In stage *i*, processor with rank *j* computes the submatrix 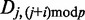. If *P*-value is even, the submatrices computed in the last stage are duplicately assigned to two processors each, due to symmetry (shown in dark squares in Figure 1). In this case, half the submatrix is computed on each processor to avoid redundant computation.

In the first stage (*i* = 0), the expression profiles of the genes corresponding to rows (row genes) and columns (column genes) of the assigned submatrix are the expression profiles initially assigned to the same processor. The row gene profiles needed remain unchanged throughout the stages. For columns, the profiles needed by processor with rank *j* in stage *i* are the same profiles used by processor with next rank (*j* + 1) (for rank *p* – 1 next processor is 0) in previous stage (Figure 1). This circular left shift pattern communication is commonplace in parallel computing.

We compute the MI-value between a pair of gene expression profiles using the B-splines method proposed by Daub *et al.* (35) which runs in *O*(*m*) time. The statistical significance of each MI-value is assessed using permutation testing—by generating *P* random permutations of one of the expression profiles, recomputing the MI-value based on each permutation, and accepting the original MI-value as significant only if it is higher than at least a fraction (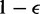) of the permutations tested (for a fixed, small 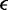). Permutation testing is computationally expensive and previous MI-based network inference methods did not employ it directly. We developed an algorithm to perform direct permutation testing collectively for all pairwise gene MI computations such that the overall complexity is reduced by a factor of 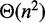 as described next.

### Efficient permutation testing through rank transformation

Let 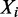 denote the vector of gene expression observations for gene *i* and 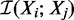 denote the MI between vectors 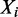 and 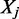. It is well known that MI has the property of being invariant under homeomorphic transformations (36,37):
(1)
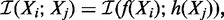

for any homeomorphisms *f* and *h*. Consider replacing the vector of observations for gene *i*, i.e. 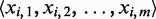 with the vector 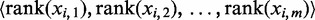, where 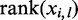 denotes the rank of 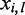 in the set 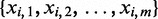, i.e. we replace each gene expression value with its rank in the set of observed expression values for the gene. The transformation, which is termed ‘rank transformation’, while not continuous, is considered a good approximation to homeomorphism (37). Instead of computing MI of pairs of gene expression vectors directly, we equivalently compute the MI of their rank-transformed counterparts. With this change, each gene expression vector is now a permutation of 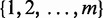. Therefore, a permutation 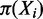 also corresponds to some permutation of the observed vector 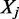 for any other gene *j*. Thus, each permutation test is a valid test for all 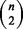 pairs of observations. Therefore, one can use a total of *P* permutation tests, instead of *P* permutation tests for each pair, reducing the work in permutation testing by a factor of 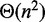. Moreover, with this change estimation of marginal probabilities required in computing MI depends only on the number of observations, and thus can be computed collectively once for all expression profiles.

There are important side benefits to our approach with regards to both quality and computational efficiency: while permutation testing of a pair by itself is an agreed upon statistical technique, evaluating the significance of 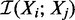 with respect to all 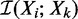 (for 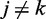) is important to extract the more prominent interactions for a gene. This is naturally incorporated in our scheme as a fixed number of permutation tests are conducted on each pair, and then collectively used to assess the statistical significance of every pair. Computational efficiency is obtained by exploiting the observation that each expression vector is a permutation of 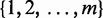. As rank-transformed data consist of equispaced observations, it also improve the performance of a majority of MI estimators.

### Removing indirect interactions in parallel

As in (6), we use DPI to remove indirect interactions, except that we developed a parallel method to do so. DPI states that if three random variables 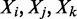 form a Markov chain in that order (i.e. conditional distribution of 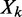 depends only on 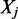 and is independent of 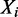), then 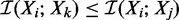, which also implies that 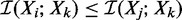. These inequalities can be used to discard indirect interactions: each time the pair 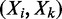 satisfies both inequalities as described above, the corresponding edge between gene *i* and gene *k* is removed from the network.

To decide whether a given edge *D*[*i, j*] is the result of indirect interaction, complete information about rows *i* and *j* are needed. As matrix *D* is stored row-wise, we need to stream row *j* to the processor responsible for row *i*. Moreover, because matrix *D* is symmetric, it is sufficient to analyze its upper (or lower) triangular part. This is achieved in *p* – 1 communication rounds, where in round *i* only processors with ranks 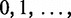 *p* − *i* participate in communication and processing. The worst-case parallel run-time of this phase is 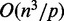-value. The worst case is overly pessimistic as DPI needs to be applied only to current existing edges, and the network is expected to be significantly sparse. In computing our whole-genome network, we found that this phase takes <1% of the total time.

### Run-time analysis

Although a worst-case run-time analysis indicates DPI application to be the computationally dominant phase, we found that over 99% of the run-time is accounted for in computing the MI-values (inclusive of permutation tests) for the whole-genome network. The run-time behavior is 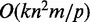, where *n* is the number of genes, *m* is the number of observations, *P*-value is the number of processors (or cores), *k* is the number of permutation tests conducted per edge and 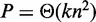 is the number of permutation tests used to evaluate the statistical significance of each edge. The storage required is 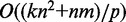. Both the run-time and storage reduce linearly with the number of processors used, enabling our method to scale to very large networks and gene expression profiles by utilizing larger scale parallel computers.

### Subnetwork extraction

We developed a method that takes genes from a partially characterized pathway (or cellular process) as input and uses the whole-genome network to predict potential candidate genes that might play a role in the process. This is achieved by extracting a subnetwork containing the given pathway genes. Our method is based on ideas drawn from ranking of web pages for relevance using random walks on the world wide web graph (38). It is similar to prior applications of this strategy in prioritizing gene lists based on network topology (29–32). However, we go one step further and incorporate the genes one by one in rank order until the subgraph induced by the set of seed genes and incorporated genes together forms one connected component. The induced subgraph is then returned to the user as the extracted subnetwork.

The method works as follows: we first convert our network *N* into an equivalent first-order Markov chain by assigning transition probability 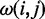 to each edge (*i, j*) as follows:

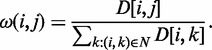

Taking the given seeds, we then rank all genes in the network in terms of their relative importance to the seed genes. We determine rank *R*(*j*) of gene *j* iteratively using:

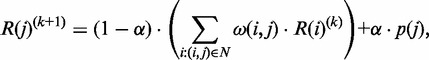

where 
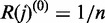
, *P*-value(*j*) specifies preference for node *j* and 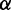 denotes the probability of ‘returning’ to one of the seed nodes. A value of 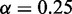 is used for the extracted subnetworks presented in the article. The function *P*-value(*j*) captures prior knowledge about the partially characterized pathway by forcing the ‘return’ to be one of the known genes. Its value is set to 1/*t* for each known gene in the pathway where *t* is the number of known genes, and set to 0 otherwise. The ranking process is performed iteratively until it stabilizes to within a specified threshold. As the final ranking corresponds to steady-state distribution of the underlying Markov chain, the network *N* must be connected and aperiodic. In our experiments, 20–25 iterations proved sufficient for convergence. Once ranking of all genes is identified in this manner, they are added one by one to the partially characterized pathway until the subnetwork induced by these genes forms one connected component. The induced subnetwork is then returned as the prediction. The subnetwork extraction method is serial. As the entire process took only a few seconds on a commodity workstation even on the genome-scale network we generated, there is little practical advantage to be gained by pursuing parallelization of this method.

### Software availability

Based on the methods described above, we developed software packages TINGe and GeNA. TINGe is a parallel program implemented in C++ and MPI and made available as open source. The accompanying program GeNA is implemented as a Cytoscape plugin. Note that GeNA can be used on a network created by any inference method and has standalone applicability. Both TINGe and GeNA are available at the website http://aluru-sun.ece.iastate.edu/tinge.

## RESULTS AND DISCUSSION

### Quality assessment of the parallel method

As a first step to evaluate the quality of predicted interactions, we used well-regarded benchmarks (39) and DREAM4 network inference challenge (14). To provide a context for the results obtained from these tests, we compare our method with two well-established network inference methods relevant to our work: ARACNe (Algorithm for the Reconstruction of Accurate Cellular Networks) (6), which is based on MI, and GeneNet (4), which is based on GGM and was previously used for *Arabidopsis* network inference.

**Figure 2.**
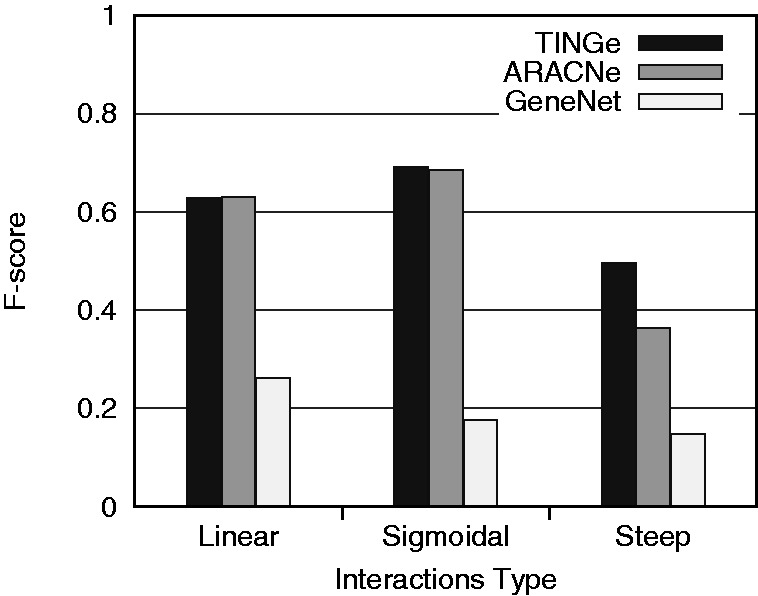
Comparison of TINGe with ARACNe and GeneNet on synthetic data using SynTReN.

We performed a set of experiments using synthetic data generated by the SynTReN package (39). SynTReN generates realistic reference network topologies by sampling from an input network while preserving its essential topological properties. For our purpose, we selected the biologically validated Yeast network extracted from the BIND database (40). We considered three types of interactions, in which expression of a gene is a linear, sigmoidal or steep function of the expression of its regulators, posing successively increasing difficulty for the three inference methods. Using default SynTReN parameters, we generated networks with 100 and 500 genes for which 200 expression observations were simulated, repeating 10 times for each type of interaction. We measured the average precision (percentage of correct edges among all edges inferred) and recall (percentage of correct edges predicted) of each inference program and compared them using the *F*-score measure defined as: 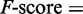

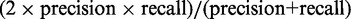
. Figure 2 summarizes results from the comparison of the three inference methods. TINGe performs similar to ARACNe for linear and sigmoidal interactions, and slightly better for steep-like functions. Note that TINGe results need not be identical to ARACNe due to the different approach used in estimating MI, and the application of permutation testing to select MI threshold values. In all three cases, TINGe significantly outperforms GeneNet (Supplementary Table S2).

**Figure 3.**
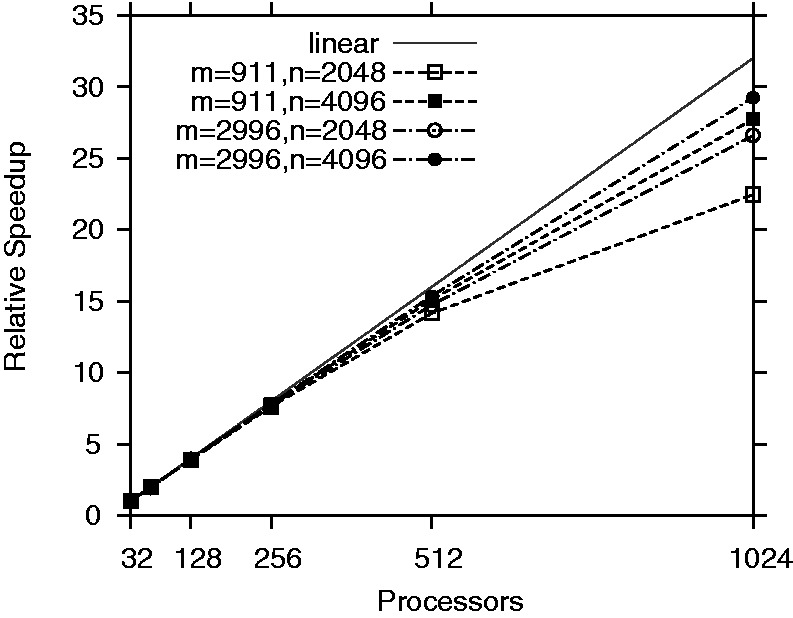
Scalability of TINGe for datasets with different numbers of genes *n*, and different numbers of expression observations *m*.

Although conceptually similar to other information theory-based methods (6,7), TINGe combines parallel processing with rigorous statistical testing and is unique with respect to the way MI threshold is obtained. Moreover, due to algorithmic innovations, TINGe is roughly five to six times faster than ARACNe (MI-based method) even on a sequential basis (Supplementary Table S2). With respect to this improved sequential performance, TINGe exhibits near linear improvement in run-time with the number of processors used, enabling our method to scale to very large networks and gene expression profiles by utilizing either commodity clusters or parallel computers (Figure 3 and Supplementary Table S3).

SynTReN is a well-established benchmark; however, it has been argued that the expression kinetics model it uses is too simplified. Therefore, we further validated TINGe using the DREAM4 In-Silico Network Challenge (14), designed to test network inference methods using realistic gene expression models, including network topology and expression kinetics. Although DREAM4 was envisioned as a competition to assess network inference methods at that point in time, we consider it a useful benchmark to evaluate our new method and how it would stack up against the methods assessed by the competition, meaningful because of the recency of the competition. We used the ‘Size100_Multifactorial_Undirected’ benchmark that provides synthetic data that resemble an aggregation of multiple microarray experiments. The benchmark consists of five microarray datasets generated using the GNW package (41) for five different synthetic networks to be predicted. The quality of predictions is measured using the area under the precision versus recall (AUPR) curve, and the area under receiver operating characteristic (AUROC) curve, taking into account all edges in tested networks. The statistical significance of inferred networks is obtained by comparing them with a null model built from a large number (10 000) of completely random predictions. The final score is based on the overall quality of all five reconstructed networks (a log-transformed ‘average’ of the two overall AUROC and AUPR *P*-values, which are geometric means of individual *P*-values). In our tests, TINGe obtained 34.7486 total score with 14.5238 AUROC score and 54.9733 AUPR score, with the corresponding *P*-values being 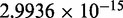 and 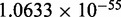, respectively. This score places TINGe between the best (score 37.299) and the second best (score 31.645) performing DREAM4 competitors in this category (http://wiki.c2b2.columbia.edu/dream/results/DREAM4). Receiver operating characteristic curves summarizing TINGe performance for the test networks are shown in Supplementary Figure S1.

### Reconstruction of the *Arabidopsis* whole-genome network

*Arabidopsis* network inference has hitherto consisted mainly of targeted studies such as modeling the isoprenoid gene network (5), transcription factor-induced network (42) and three recent works aimed at whole-genome network inference (2,3,15). A Pearson correlation network of 6206 genes from 1094 microarrays was reported by Mao *et al.* (2). Ma *et al.* (3) constructed a network of 6760 genes from 2045 microarrays using Pearson correlation and GGM. This was constructed piecemeal by considering 2000 randomly selected genes at a time and using 2000 such samples to cover the network to deal with computational limitations. Even so, each round of 2000 network inferences took ∼4 days (3). Both networks span only 25% of *Arabidopsis* genes and assume linear models of gene co-expression. The third network resource of *Arabidopsis* is the AraNet (15). This is not directly comparable to networks inferred by gene expression data alone—what sets this network apart is the integration of 24 types of ‘omics’ data from various organisms, one of which is gene expression data. However, much of AraNet’s predictive power for gene interactions in the network is dependent on plant-derived data. For reconstructing interactions based on *Arabidopsis* expression data, AraNet uses Pearson correlation. To date, it has not been possible to directly construct genome-scale networks from thousands of gene expression profiles using sophisticated non-linear approaches such as MI. In this article, we report on the reconstruction of such a whole-genome network of *A. thaliana*.

**Figure 4.**
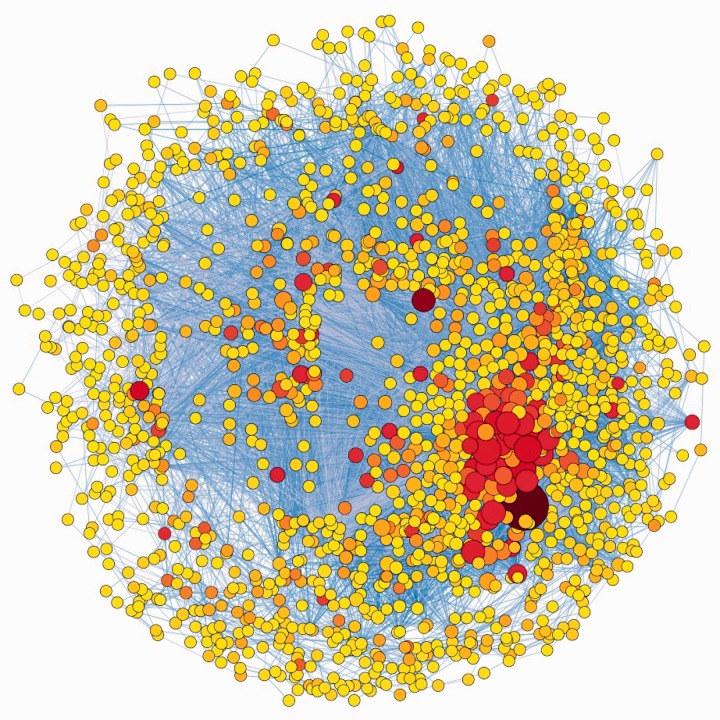
A partial rendering of the *Arabidopsis* whole-genome network. The illustrated network represents a union of all the shortest paths between each pair of the top 5% of the hubs in the whole-genome network (Supplementary Table S4). It contains 1556 genes and 22 073 interactions. The network topology is displayed using Cytoscape with the size of a node proportional to its degree and the intensity of its color proportional to its betweenness centrality. The largest and the darkest node in the bottom right-hand corner of the figure is PMDH2, a gene involved in photorespiration.

We collected 3546 expression profiles on the GeneChip Affymetrix ATH1 Genome Array platform covering a range of cellular and physiological states for network reconstruction. The number of expression profiles were determined solely based on availability from multiple public repositories and their relevance to network inference, and the same GeneChip requirement was enforced to make it reasonable to evaluate expression levels across different experiments. Collective analysis of data generated from different experiments in various laboratories around the world poses a challenge in data preparation due to technical, experimental and laboratory-to-laboratory variations (43). We also found network quality to be critically dependent on statistical analysis and enforcement of rigorous quality control measures. After much experimentation, we evolved specific measures for quality control, statistical normalization and filtering of data, and annotation of the genes (see ‘Materials and Methods’ section). Following these measures, 3137 GeneChips and 15 495 genes remained for network construction. Using TINGe, we constructed the 15 495 gene network in 9 min on a 1024-core computer cluster (Figure 4 and Supplementary Table S4). Even though this is the largest number of gene expression profiles collectively analyzed over any prior work on *Arabidopsis* network inference, our parallel method can easily scale to include all of the genes in the *Arabidopsis* genome and to many more gene expression datasets should they become available. The number of microarray experiments currently available in *Arabidopsis* public databases is not sufficient yet to allow for a complete model of the *Arabidopsis* transcriptome. The genes missing in our network did not have the dynamic range of expression needed to derive statistically meaningful assessments.

An advantage of fast network reconstruction in the range of minutes to a few hours is that it enables experimentation—testing of various statistical protocols for robust network inference, exploring parameter space, etc. We have certainly taken advantage of this to iteratively tune the computational and statistical methods, guided by continual evaluation grounded in biological knowledge, prior to finalizing the inferred genome-scale gene network. Furthermore, fast turnaround times enable deploying statistical approaches such as bootstrapping. To infer a gene network comprising of tens of thousands of genes in acceptable time limits, TINGe should be executed on a multiprocessor system such as a cluster. Nevertheless, in many cases, this will be a one-time effort, and it should be noted that all subsequent network analysis can be executed on a regular desktop computer. Finally, TINGe can run on single-core in which case it can be used to reconstruct networks of smaller size. One limitation of TINGe is that it does not infer directionality. This limitation is common to all MI-based methods, and it is further shared by all pairwise correlation-based methods.

### Network properties

The TINGe generated *Arabidopsis* network consists of 132 762 interactions giving it a density of ∼0.001. The average node degree (average number of interactions per gene) is 17, and the diameter (shortest path length between two farthest genes) is 10 (Table 2).

**Table 2.** Summary of network properties

Number of nodes	15 495
Number of edges	132 762
Density	0.00111
Average node degree	17.13
Diameter	10
Characteristic path length (*L*)	3.96
Average clustering coefficient (*C*)	0.23
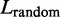	3.4
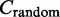	0.00013
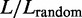	1.17
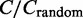	1782.56

The characteristic path length (*L*) represents the length of the average shortest path computed over all pairs of genes in the network, and *C* is the average clustering coefficient computed over all nodes. 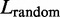 and 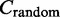 are the expected values of *L* and *C* for a completely random network with the same number of nodes. Proximity between *L* and 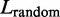, and the large discrepancy between *C* and 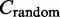 indicate small world property of the *Arabidopsis* network.

**Figure 5.**
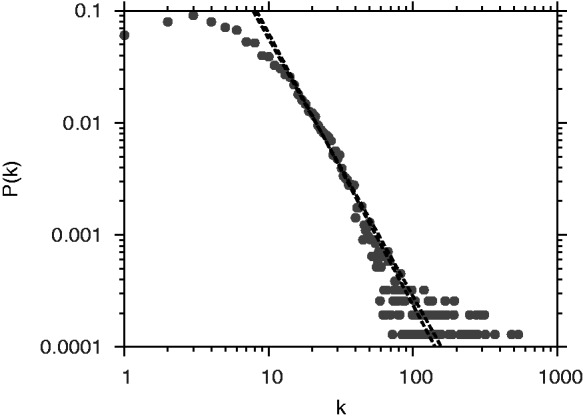
Scale-free nature of the *Arabidopsis* whole-genome network. Node degree distribution in the network. *X*-axis is the node degree and *Y*-axis represents the probability of a node with a given degree.

A network is considered scale-free if the distribution of node degrees follows a power-law distribution: 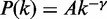, where *A* is a constant, 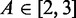, and *P*(*k*) is the fraction of nodes with degree *k* (44). It follows that a log–log plot of *P*(*k*) versus *k* should be a straight line with slope 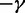. Similarly, a log–log plot of the cumulative distribution function (fraction of nodes with degree 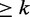) should be a straight line. It is readily observed that the *Arabidopsis* whole-genome network exhibits scale-free property by the proximity of the data to the best-fit straight line for both degree distribution and cumulative degree distribution (Figure 5 and Supplementary Figure S2). In addition, the slope of the straight line in Figure 5 is equal to 2.29, in line with Barabasi and Oltvai’s classification of scale-free networks (44). Furthermore, the characteristic path is comparable to and the average clustering coefficient is significantly higher than what is expected in a random graph with the same number of nodes and the same average node degree, indicative of the small-world property (45) (Table 2).

There are 13 323 genes with node degree higher than 2 in the *Arabidopsis* whole-genome network. Of these, the 591 genes with 50 or more interactions collectively account for 61 550 interactions (47%) in the network (Supplementary Table S4), and control a wide range of essential cellular functions. Based on TargetP subcellular localization (http://www.cbs.dtu.dk/services/TargetP) and Fisher’s exact test, chloroplast genes are significantly over-represented among hub genes (*P*-value 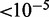). On the other hand, cytosolic and secretory genes are significantly under-represented (*P*-value 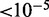). The number of mitochondrial genes is neither significantly over-represented nor significantly under-represented among hub genes (*P*-value = 0.1658). Especially noteworthy, the top 10 hubs in the list (*AT5G09660*, *AT2G46820*, *AT1G70760*, *AT3G23700*, *AT1G67740*, *AT3G55800*, *AT1G32060*, *AT1G14150*, *AT1G76450* and *AT1G68010*) are all associated with photosynthesis and related processes such as photorespiration and have a node degree >1000.

The occurrence of photosynthesis genes as major hubs is not uncommon and has been reported previously for *Arabidopsis* networks (2). Chloroplast metabolism, and in particular photosynthesis, plays a crucial role in plant survival and fitness. Our studies thus underline the importance of this process in the plant cell. Note that the generated network is also influenced by sampling bias reflecting the experimental conditions under which the microarray experiments were conducted. Many of the experiments in the microarray databases are related to development, and various stress conditions, which ultimately affect the photosynthetic process. The microarray data may also lack sufficient perturbations for the dynamic expression changes of the ‘missing hubs’ to be recorded and hence, some other important hubs/genes could have been filtered out from the network.

Many of the genes with the highest node degrees are also among those with the highest centrality scores (measures the frequency with which a gene appears on shortest paths between all pairs of genes). For instance, 7 of the top 10 hubs (*AT5G09660*, *AT2G46820*, *AT3G23700*, *AT1G70760*, *AT1G67740*, *AT1G14150* and *AT1G68010*) also have high centrality values (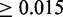). The centrality–lethality rule, although less pronounced for higher organisms, suggests that these hubs are essential for proper functioning of cellular processes (46). Consistent with this hypothesis, mutants for five of these seven genes with high centrality values (*AT2G46820*, *AT3G23700*, *AT1G70760*, *AT1G67740* and *AT1G14150*) show reduced growth and/or conditional lethal phenotypes (47–50).

### Assessment of functional modularity

Genes associated with similar biological functions form functional modules of tightly interacting genes (3,44,51,52). Therefore, to assess biological validity of the *Arabidopsis* network, we first investigated association strength among six selected sets of genes, each annotated with a different biological process (Table 3). List(s) of genes for each of these processes was obtained from the *Arabidopsis* TAIR website (Supplementary Table S5). For each of these processes with its associated set of genes, we created the induced subgraph consisting of only those edges from the whole-genome network that connect genes from this set. We then computed the number of connected components in the resulting subgraph, which should be small for a tightly connected functional module. To assess the statistical significance of the number of connected components found, we obtained the null distribution by generating 102 400 random networks by shuffling genes in our *Arabidopsis* network and repeating the subgraph extraction and connected component analysis. The low *P*-values reflect functional partitioning of genes at significantly higher rates than expected by chance and confirm strong modularity for plant cellular processes in the network (Table 3 and Supplementary Figure S3). These results further show that genes directly involved in response to stress and external stimuli such as photosynthesis, heat shock and cold stress are more significantly co-expressed when compared with those less related to stress responses such as cell cycle and brassinosteroid metabolism.

**Table 3.** Assessment of functional modules

Process	Genes	Components	*P*-value
Photosynthesis	85	7	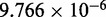
Heat shock response	30	14	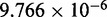
Cold response	22	9	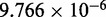
Phenylpropanoid metabolism	72	50	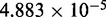
Cell cycle	26	19	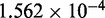
Brassinosteroid metabolism	24	20	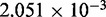

Interactions between a given set of genes (number of genes) known to be involved in a biological process were verified for functional modularity. Number of components is the number of connected components in the subnetwork induced by the input genes. *P*-value is the probability of a given number of connected components in a random network with the same number of nodes and the same node degree distribution as the input network.

We also measured the interaction strengths between genes linked to various plant organelles and observed strong organelle type-specific co-expression patterns (*P*-value 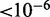 for chloroplast-targeted genes; *P*-value 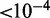 for mitochondrial genes and *P*-value 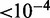 for golgi body genes) in *Arabidopsis* (data not shown). Such results also confirm metabolic compartmentation of genes for organelle-specific functions in multicellular organisms and are consistent with a priori biological knowledge (53). The finding that the whole-genome network is modular and shows biological significance provided further motivation for the identification of candidate genes and for assigning genes to cellular processes and metabolic pathways.

### Extraction of subnetworks

Although methods for network analysis and subnetwork extraction have been developed previously [see, for example (2,18,51)] they have generally been applied to networks of moderate size. As TINGe allows networks built at whole-genome scale with significantly more number of gene expression profiles, it is important to study the effectiveness of such approaches when applied at the larger scale. A general problem we found when experimenting with multiple methods is that the high connectivity of the whole-genome network resulted in subnetworks that are too large to be of practical value. For instance, the simple guide-gene approach of taking neighbors up to two edges away from the given set of seed genes (3,54) resulted in a few thousand potential candidate genes, too large to experimentally verify. Such methods also fail to take into account significance of the interactions and/or proximity to multiple guide genes. To overcome this problem and facilitate biological hypothesis testing, we developed the subnetwork analysis tool GeNA. GeNA is built by adapting the successful approach in the web search context of ranking web pages for relevancy by collectively analyzing the links between them. We developed a similar method to rank each gene in the whole-genome network, but specific to its relevance to a set of given seed genes and taking the strength of MI interactions into account. We then identify the minimum number of highest ranked genes needed to build a connected subnetwork containing the seed genes, and output this subnetwork. Although GeNA exploits the MI-values of the inferred network, it can be applied as a standalone tool on networks generated by any other inference method, and even in the absence of information on the strength of interactions (by setting them all to an equal value).

**Figure 6.**
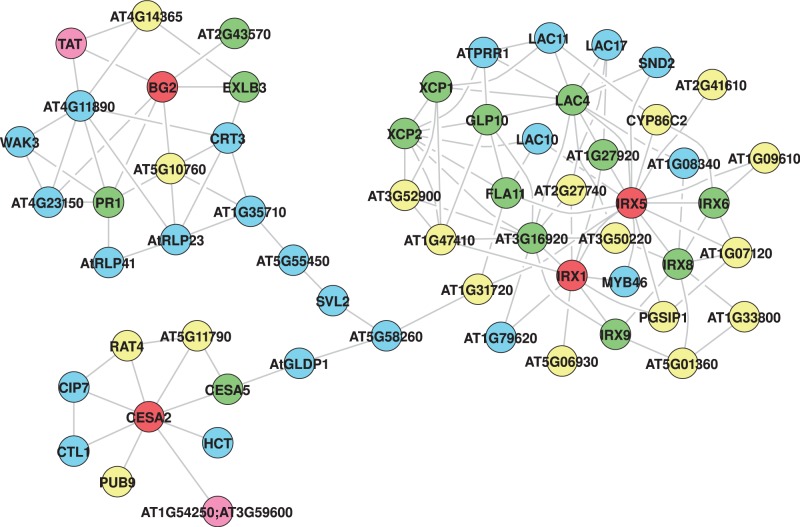
Cellulose subnetwork. Red—seed genes; green—genes sharing the same GO category as the seed genes; blue—genes with associated functions; pink—genes of interacting pathways and yellow—unclassified genes.

**Figure 7.**
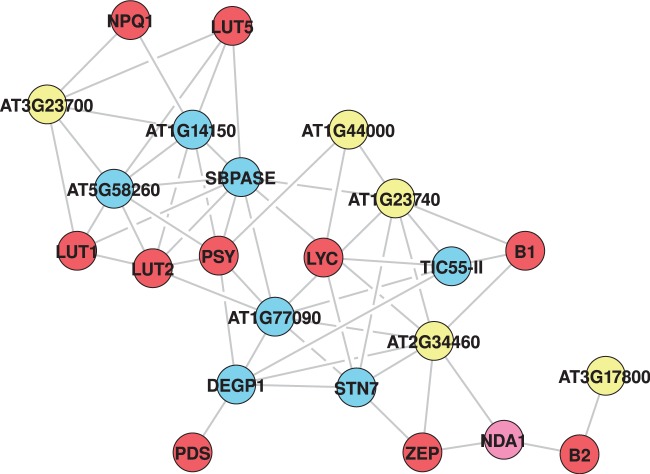
Carotenoid subnetwork. Color coding as given in Figure 6.

**Figure 8.**
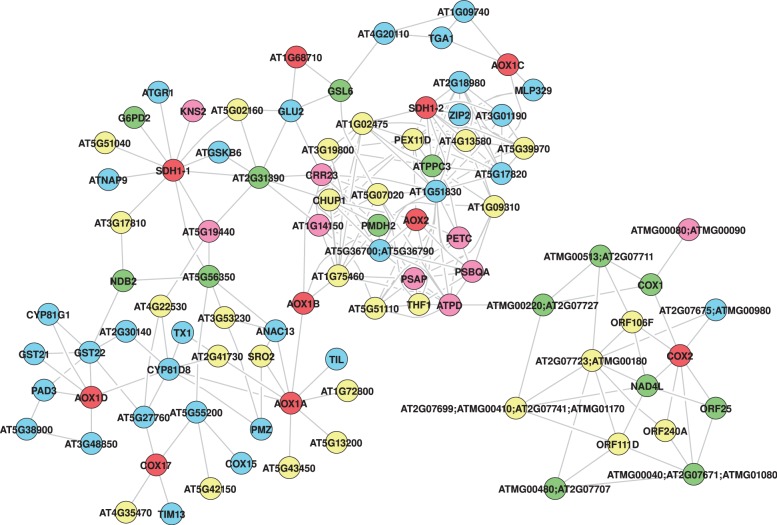
Aerobic respiration subnetwork. Color coding as given in Figure 6.

Using subnetworks of three example pathways (Figures 6–8), we show general utility of this approach in extracting context-specific subnetworks from the *Arabidopsis* whole-genome network. The GeNA extracted subnetworks contain additional genes linked to the same pathway or cellular process (green color nodes), and incorporate interactions between genes of known and unknown function. In this way, GeNA can be used to make functional predictions of unclassified genes, and for revealing insights into crosstalk between various cellular processes. Annotation and functional categorization of genes in subnetworks is based on the *Arabidopsis* MIPS classification scheme (http://mips.helmholtz-muenchen.de/proj/funcatDB), and/or TAIR gene ontology (GO) annotations (http://www.arabidopsis.org). In addition, functional enrichment analysis was done using GOMiner (http://discover.nci.nih.gov/gominer/index.jsp) (Supplementary Table S6). The quality of extracted subnetwork is sensitive to the set of seed genes and improves with increase in number of known genes and/or knowledge of important genes (such as at branches of the given pathway).

### Cellulose biosynthesis

The GeNA extracted cellulose subnetwork is highly compact in terms of functional categories given that only four known cellulose biosynthesis genes (*BG2*, *CESA2*, *IRX1* and *IRX5*) were provided as seed genes for subnetwork extraction (Figure 6). We find that in addition to unclassified genes, genes with only three major functional categories were extracted from the whole-genome network in the subnetwork; cell wall metabolism, lignin metabolism and kinases (Supplementary Table S7), all of which are associated with biogenesis and functioning of plant cell walls. These results are consistent with prior studies demonstrating significant co-expression of genes mediating cellulose biosynthesis (52). Moreover, network topology reveals distinct clustering of genes for primary cell wall biosynthesis (*CESA2* and *CESA5*), secondary cell wall biogenesis (*CESA8* (*IRX1*) and the other IRX group of genes) and kinases (55,56). This type of grouping allows for improved functional prediction(s) of unclassified genes by assigning genes not just to individual pathways or processes but also to different branches of the pathway. For example, based on the distinct grouping, it is now possible to hypothesize that *PUB9* and *AT5G11790* function in primary cell wall biogenesis while others including *AT2G41610*, *AT1G07120*, *CYP86C2* and *AT3G50220* are related to secondary cell wall biogenesis.

In addition to incorporating wall-associated kinases (WAK and WAKL) which function in cell expansion and signaling (57), the subnetwork also includes a new class of kinases (Figure 6). These kinases (*AT1G35710*, *AT1G79620*, *AT4G11890* and *AT4G23150*) lack the calcium binding EGF-like domain present in WAKs and WAKLs and contain the leucine rich repeat domains absent in WAKs and WAKLs. Thus, TINGe together with GeNA provide new insights into putative function(s) of some of these orphan receptor kinases that perhaps define novel signaling pathways regulating cell wall biogenesis and/or function.

### Carotenoid biosynthesis

The carotenoid subnetwork was constructed around 10 seed genes (*PSY*, *PDS*, *LYC*, *LUT2*, *LUT5*, *LUT1*, *B1*, *B2*, *NPQ1* and *ZEP*) known to function in the carotenogenesis pathway (Figure 7 and Supplementary Table S7). This pathway is a branched pathway, with the two branches of the pathway leading to the formation of lutein and carotenes, respectively (58). Similarly, we find branch-specific expression patterns for carotenogenesis genes; genes involved in lutein biosynthesis (*LUT1*, *LUT2*, *LUT5*) are more closely linked in the network when compared with genes of the carotene branch of the pathway (*LYC*, *ZEP*, *B1* and *B2*), while the gene common to both branches (*PSY*) appears to form a connecting bridge (Figure 7). The subnetwork incorporates several genes encoding proteins with critical functional roles in photosynthesis. The high co-expression significance of carotenoid genes with genes mediating photosynthesis is consistent with the known function of carotenoids in plants, and gives high confidence in the accuracy of network predictions. In addition, several genes of unknown function are associated with genes of carotenogenesis, all of which are predicted to be targeted to the chloroplast where carotenoid biosynthesis occurs. It is noteworthy that *NDA1*, which codes for an inner mitochondrial membrane protein is included in the subnetwork, thus suggesting a role for *NDA1* in interorganellar signaling between the chloroplast and mitochondria (59), perhaps through the manipulation of genes in the carotenoid biosynthesis pathway.

### Aerobic respiration

Analysis of aerobic respiration subnetwork revealed that many of the genes (including the seed genes) function in glycolysis, fermentation, tricarboxylic acid cycle and electron transport (Figure 8 and Supplementary Table S7) (60). Other major functional categories include stress response and transport. *AOX1A* and *AOX1D* (two of the seed genes) are known to be some of the most stress responsive proteins among the mitochondrial proteins (61), and genes in the stress response category are involved in combating oxidative stress. Genes in the transport category are predicted to be either involved in mitochondrial (*AT1G61570*, *AT3G48850* and *AT5G55200*) or in intracellular transport of substrates and electrons. The subnetwork also includes genes involved in photosynthesis, and crosstalk between the respiratory pathway and photosynthesis is a well-known phenomenon in plants (60). Thus, analysis of the whole-genome network reveals genes crucial for signaling between these two pathways.

To similarly predict novel members of various other metabolic pathways, we used GeNA to extract subnetworks for all pathways listed in the *Arabidopsis* AraCyc database (http://www.arabidopsis.org/biocyc) (62). For each pathway in the database, only genes that have experimental evidence were taken to be seed genes, and analysis was restricted to pathways with at least three seed genes. Of a total of 446 pathways obtained (AraCyc 8.0 Release—April 2011), 241 met the criteria. The list of the resulting 241 metabolic pathways analyzed, corresponding seed genes, additional genes extracted by GeNA and gene ranks are provided at http://aluru-sun.ece.iastate.edu/tinge for further exploration.

## CONCLUSIONS

High-throughput data-driven systems biology is computationally intensive, and by all indications, the data explosion will only continue to grow over the next few years. Dogged by computational and memory issues, network inference methods are forced to compromise on one or more of the following: number of genes, number of experiments, method for inferring gene interactions and method for inferring their statistical significance. The primary goal of developing our parallel method and resulting software TINGe is to infer gene networks at the genome scale for any given organism using all available gene expression profiles. Our method does not place limitations on the number of genes or expression profiles and chooses a suite of rigorous statistical and computation protocols which have not been collectively employed before, even at a smaller than genome scale. Thus, the scale of computation achieved and represented in this study is beyond the reach of current methods. Although TINGe is accompanied by software for effective quality control and normalization across diverse microarray experiments, it is primarily a parallel method to infer networks from gene expression values, and as such can process gene expression profiles generated by other means such as RNA-seq.

TINGe can be used for directly constructing high-quality networks, or it can be used as a component along with other types of data in building probabilistic networks such as AraNet (15). Pop *et al.* (63) build a compendium of tissue-specific, developmental stage-specific and process-specific *Arabidopsis* networks using Bayesian classifiers for heterogenous data integration, including a small number of microarray experiments. As several gene interactions are context specific, such a compendium of networks is of great value in elucidating comprehensive functional relationships. Such efforts can be greatly aided by TINGe. One can classify microarray experiments as desired (tissue-specific, stage-specific, etc.) and easily build numerous functional networks using TINGe at unprecedented scale and speed. Although we use *Arabidopsis* as an example for our network inference method, it is possible to similarly generate genome-scale gene networks and/or gene regulatory networks of other complex organisms using TINGe and make further inroads through comparative network analysis.

## SUPPLEMENTARY DATA

Supplementary Data are available at NAR Online: Supplementary Tables 1–7 and Supplementary Figures 1–3.

## FUNDING

The US National Science Foundation [CCF-0811804]; a Swarnajayanti Fellowship from the Department of Science and Tehcnology of the Government of India. Funding for open access charge: Research funding available to S.A.

*Conflict of interest statement*. None declared.

## Supplementary Material

Supplementary Data
