# Supplementary material for: Reverse engineering and analysis of large genome-scale gene networks
Source: Nucleic Acids Res. 2012 Oct 5;41(1):e24. doi: 10.1093/nar/gks904 (PMC3592423; doi:10.1093/nar/gks904)
Supplement: Supplementary Data [file supp_41_1_e24__index.html]

Reverse engineering and analysis of large genome-scale gene networks — Reverse engineering and analysis of large genome-scale gene networks — Supplementary Data 

# Reverse engineering and analysis of large genome-scale gene networks

## Supplementary Data

files

**Files in this Data Supplement:**

- Supplementary Data - pdf file
- Supplementary Data - xls file
- Supplementary Data - xls file
- Supplementary Data - xls file
- Supplementary Data - xls file
