# Supplementary material for: Reverse engineering and analysis of large genome-scale gene networks
Source: Nucleic Acids Res. 2012 Oct 5;41(1):e24. doi: 10.1093/nar/gks904 (PMC3592423; doi:10.1093/nar/gks904)
Supplement: Supplementary Data [file supp_gks904_nar-00583-met-n-2012-File017.pdf]

Supplementary Figures and Tables

Supplemental Figure S1. Receiver operating characteristic curves illustrating the performance of TINGe on the five different DREAM4 test networks.

Supplemental Figure S2. Cumulative node degree distribution in the reconstructed *Arabidopsis* whole-genome network.

Supplemental Figure S3. Probability distribution of the number of connected components observed in 102,400 random networks for each biological process. Vertical line indicates the number of connected components in the input network.

Supplemental Table S1. List of various microarray experiments obtained from different public repositories.

Supplemental Table S2. Comparison of performance and run-time of three network reconstruction methods on synthetic data from SynTReN.

Supplemental Table S3. TINGe runtime in seconds for different numbers of genes n, and different number of expression observations m

Supplemental Table S4. List of genes included in the network. Information about the node degree, betweenness centrality, clustering coefficient and annotation is provided for all genes in the network.

Supplemental Table S5. List of genes used for biological verification of gene-gene interactions in the *Arabidopsis* whole-genome network. Each sheet in the file contains genes for a particular cellular process. Genes for each process were downloaded from the Arabidopsis TAIR website (http://www.arabidopsis.org).

Supplemental Table S6. Functional enrichment analysis of genes in GeNA extracted subnetworks using GOMiner. For each GO term, the total number of genes with that functional category in the *Arabidopsis* whole-genome network and the GeNA extracted subnetwork, respectively, are shown. The number of "in subnetwork" genes does not include seed genes.

Supplemental Table S7. List of genes used to generate subnetworks in Figures 4, 5 and 6. Information on GeneRank and functional classification is provided for each gene.

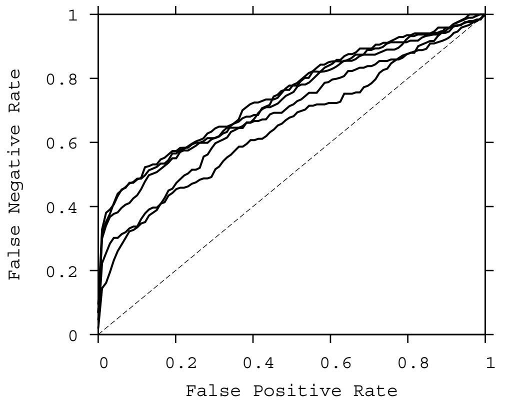

False Negative Rate
False Positive Rate
0
0.2
0.4
0.6
0.8
1

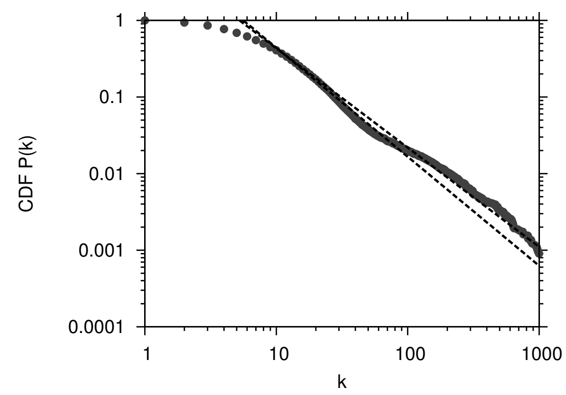

CDF P(k)
1
0.1
0.01
0.001
0.0001
1
10
100
1000
k

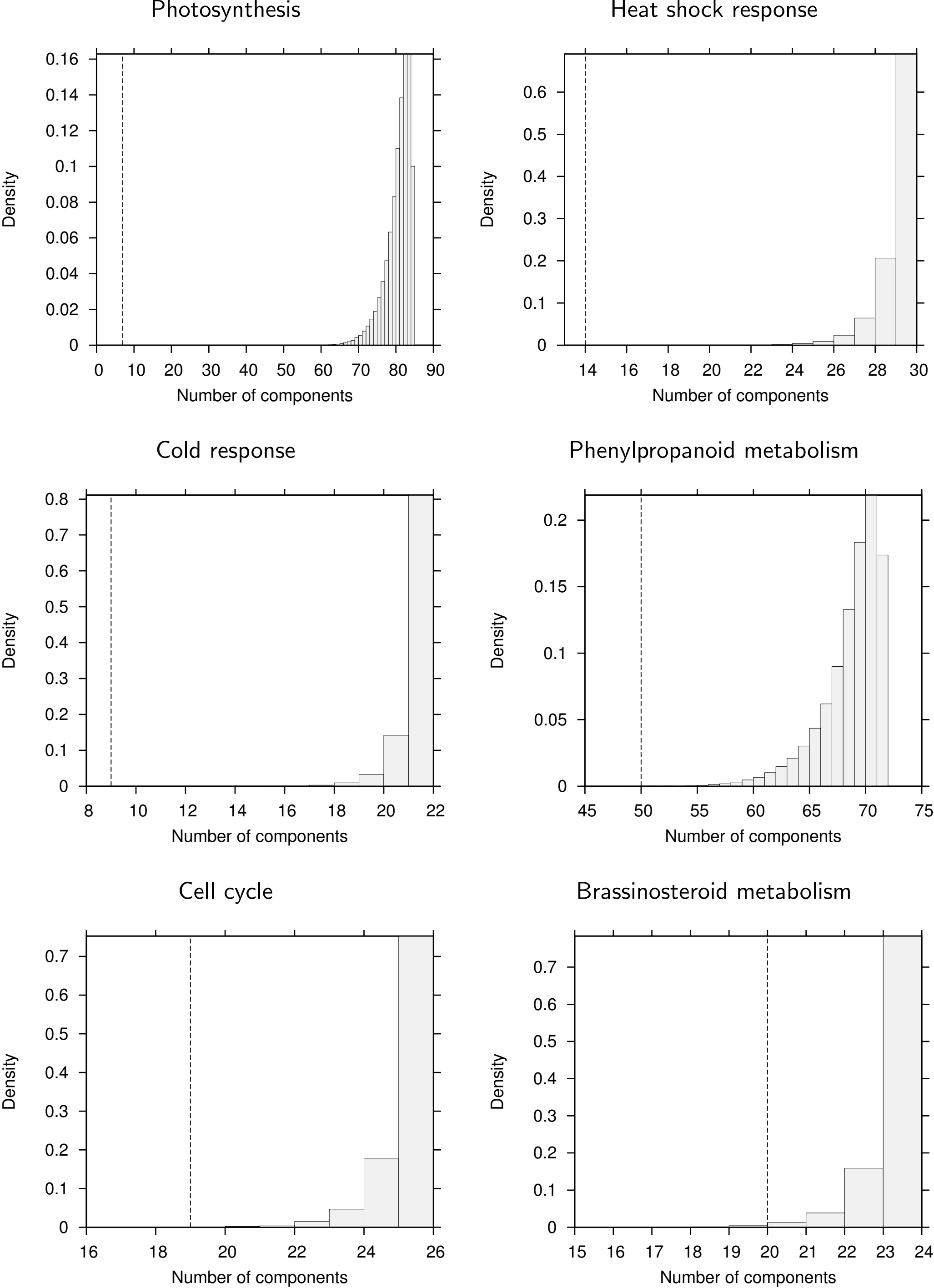

Photosynthesis
Density
0.16
0.14
0.12
0.1
0.08
0.06
0.04
0.02
0
0 10 20 30 40 50 60 70 80 90
Number of components
Heat shock response
Density
0.6
0.5
0.4
0.3
0.2
0.1
0
14 16 18 20 22 24 26 28 30
Number of components
Cold response
Density
0.8
0.7
0.6
0.5
0.4
0.3
0.2
0.1
0
8 10 12 14 16 18 20 22
Number of components
Phenylpropanoid metabolism
Density
0.2
0.15
0.1
0.05
0
45 50 55 60 65 70 75
Number of components
Cell cycle
Density
0.7
0.6
0.5
0.4
0.3
0.2
0.1
0
16 18 20 22 24 26
Number of components
Brassinosteroid metabolism
Density
0.7
0.6
0.5
0.4
0.3
0.2
0.1
0
15 16 17 18 19 20 21 22 23 24
Number of components
